# Acute Appendicitis Is Associated with Peptic Ulcers: A Population-based Study

**DOI:** 10.1038/srep18044

**Published:** 2015-12-08

**Authors:** Ming-Chieh Tsai, Li-Ting Kao, Herng-Ching Lin, Shiu-Dong Chung, Cha-Ze Lee

**Affiliations:** 1 Division of Gastroenterology, Department of Internal Medicine, Cathay General Hospital, Taipei, Taiwan; 2 Sleep Research Center, Taipei Medical University Hospital, Taipei, Taiwan; 3 Graduate Institute of Life Science, National Defense Medical Center, Taipei, Taiwan; 4 Department of Surgery, Far Eastern Memorial Hospital, Banciao, Taipei, Taiwan; 5 School of Medicine, Fu-Jen Catholic University, New Taipei City, Taiwan; 6 Department of Internal Medicine, National Taiwan University Hospital and College of Medicine, Taipei, Taiwan

## Abstract

Despite some studies having indicated a possible association between appendicitis and duodenal ulcers, this association was mainly based on regional samples or limited clinician experiences, and as such, did not permit unequivocal conclusions. In this case-control study, we examined the association of acute appendicitis with peptic ulcers using a population-based database. We included 3574 patients with acute appendicitis as cases and 3574 sex- and age-matched controls. A Chi-squared test showed that there was a significant difference in the prevalences of prior peptic ulcers between cases and controls (21.7% vs. 16.8%, *p* < 0.001). The adjusted odds ratio (OR) of prior peptic ulcers for cases was 1.40 (95% confidence interval [CI]: 1.24~1.54, *p* < 0.001) com*p*ared to controls. The results further revealed that younger groups demonstrated higher ORs for prior peptic ulcers among cases than controls. In particular, the adjusted OR for cases < 30 years old was as high as 1.65 (95% CI = 1.25~2.19; *p* < 0.001) compared to controls. However, we failed to observe an association of acute appendicitis with peptic ulcers in the ≥ 60-year age group (OR = 1.19, 95% CI = 0.93~1.52). We concluded that there is an association between acute appendicitis and a previous diagnosis of peptic ulcers.

Acute appendicitis is the most common acute surgical condition of the abdomen. In the general population, the lifetime risk of acute appendicitis is about 7%~16%^1^,^2^. The etiology of acute appendicitis remains uncertain. Obstruction of the appendiceal lumen is considered an important part of the pathogenesis of acute appendicitis. The obstruction can have multiple causes, including fecaliths, lymphoid hyperplasia (related to viral illnesses, and bacterial and fungal infections), parasites, foreign bodies, Crohn’s disease, tuberculosis, tumors, or endometriosis^3^,^4^,^5^. However, two recent large-scale studies showed that only 6.8% and 10.5% of appendicitis specimens had mechanical obstruction^6^,^7^. Furthermore, previous studies reported that in a certain portion of appendicitis cases, appendiceal lumen obstruction was due to spasms or hypertonicity of the neuromusculature at the appendicocecal juncture similar to the mechanism of pylorospasms, caused by an autonomic nervous imbalance^8^,^9^. Other studies also suggested that the frequent concurrence of pylorospasms, duodenal ulcers, and appendicitis was related to an association of direct nerve connections among the appendix, pylorus, and duodenum^10^,^11^.

Despite some studies having indicated a possible association between appendicitis and duodenal ulcers, this association was mainly based on regional samples or limited clinician experiences, and as such, did not permit unequivocal conclusions. This case-control study aimed to examine the association of acute appendicitis with peptic ulcers using a population-based database.

## Methods

### Database

Data for this case-control study were sourced from the “Longitudinal Health Insurance Database 2005 (LHID2005)”. Taiwan has been implementing its National Health Insurance (NHI) program since 1995, and the LHID2005 includes registration files and original medical claims for 1,000,000 enrollees. These 1,000,000 enrollees were randomly selected from all enrollees listed in the 2005 Registry of Beneficiaries under the NHI program (*n* = 25.68 million) by the Taiwan National Health Research Institute. Many researchers have employed the LHID2005 to longitudinally trace the utilization of medical services for these 1,000,000 enrollees. The LHID2005 also provides an excellent opportunity to explore the association of acute appendicitis with peptic ulcers. The Taiwan National Health Research Institute and independent researchers have demonstrated the representative validity of the data from the NHI program^12^,^13^. Additionally, many researchers have utilized the LHID2005 to conduct and publish studies in internationally peer-reviewed journals^14^,^15^.

This study was exempt from full review by the Institutional Review Board of the National Defense Medical Center since the LHID2005 consists of de-identified secondary data released to the public for research purposes.

### Study sample

We first selected cases by identifying 4287 hospitalized patients with a first-time principal discharge diagnosis of acute appendicitis (International Classification of Disease, Ninth Revision, Clinical Modification (ICD-9-CM) codes 540, 540.0, 540.1, and 540.9) between January 2008 and December 2012. Patients aged <18 years old (*n* = 713) were excluded in order to limit the study sample to the adult population. As a result, 3574 patients with acute appendicitis were included as cases. We further defined the date of receiving their first-time diagnosis of acute appendicitis as the index date.

For the controls, we first excluded all enrollees who had a history of acute appendicitis from the Registry of Beneficiaries since initiation of the NHI program in 1995. We then retrieved 3574 enrollees (one for every case) matched to the cases in terms of sex, age, and year of the index date. A control was selected by matching them to a given case with acute appendicitis simply on their utilization of medical services in the same index year of that particular case. We further defined the date of the first use of ambulatory care in that matched year as the index date for controls.

### Exposure assessment

We identified peptic ulcer cases based on ICD-9-CM codes 531~533. In Taiwan, when a physician suspects that a patient is affected by a peptic ulcer, he or she may provide the patient with a temporary diagnosis of a peptic ulcer in order to conduct the associated clinical examinations or lab tests for confirmation. The peptic ulcer is frequently diagnosed by gastroenterologists based upon characteristic symptoms, endoscopies, or barium contrast x-rays, etc. If a patient has a confirmed peptic ulcer diagnosis after examinations, he or she will receive routine therapy and have a second peptic ulcer diagnosis in their next outpatient visit. On the other hand, if all feasible tests have excluded the possibility of a peptic ulcer, the patient will not receive a diagnosis of a peptic ulcer again. In order to increase the validity of the diagnoses of peptic ulcers, we selected subjects who have received two or more peptic ulcer diagnoses; with at least one being made by a certified gastroenterologist. In addition, we only consider peptic ulcer cases if they have received at least two peptic ulcer diagnoses prior to the index date.

### Statistical analysis

The SAS system (SAS, Cary, NC) was used for all statistical analyses. Pearson χ^2^ tests were performed to examine differences between cases and controls by monthly income (NT$0~15,840, NT$15,841~25,000, and ≥ NT$25,001), geographical location (northern, central, eastern, and southern Taiwan), and urbanization level of the patient’s residence (five levels with 1 being the most and 5 the least urbanized). We also performed conditional logistic regressions (conditioned on sex, age, and index year) to estimate the association of acute appendicitis with previously diagnosed peptic ulcers.

Additionally, subgroup analyses were conducted to investigate odds ratios (ORs) for peptic ulcer of sampled patients by age group (18~29, 30~59, and ≥ 60 years old). We also analyzed the association between acute appendicitis and previously diagnosed peptic ulcer stratified by the presence of gastric ulcer, duodenal ulcer, *Helicobacter pylori* (*H. pylori*)-associated peptic ulcer, and non-steroidal anti-inflammatory drugs (NSAID)-associated peptic ulcer. The patients with *H pylori*-associated peptic ulcer were categorized by those who receiving *H pylori* eradication therapy. *H pylori* eradication therapy was defined as proton pump inhibitor or H_2_ receptor antagonist, plus two antibiotics (clarithromycin, metronidazole, amoxicillin, tetracycline, or levofloxacin), combined with or without bismuth. The duration of therapy was between 7 to 14 days. NSAID-associated peptic ulcer in this study was identified as those patients who had been prescribed NSAIDs for over 30 days prior to the occurrence of peptic ulcer. Furthermore, we identified gastric ulcer cases and duodenal ulcer cases based on ICD-9-CM codes 531 and 532, respectively. Statistical significance was set at *p* < 0.05.

## Results

Of the 7148 sample patients, mean ages for the total study sample, cases, and controls were 43.3 ± 17.1 ( ± standard deviation), 43.1 ± 17.1, and 43.3 ± 17.0 years, respectively (*p* = 0.656). After matching for sex, age, and index year, Table 1 shows that there were no significant differences in urbanization level (*p = *0.951), monthly income (*p* = 0.067), geographic region (*p* = 0.132), or medical history of hyperlipidemia (*p* = 0.863), congestive heart failure (*p* = 0.215), chronic renal failure (*p* = 0.736),cirrhosis (*p* = 0.106), and malignancy (*p* = 1.000) between cases and controls. In addition, there were significant differences in prevalences of comorbidities of hypertension (17.7% vs. 19.9%; p = 0.014) and diabetes (8.0% vs. 9.3%; p = 0.048) between cases and controls.

Table 2 presents the prevalence of prior peptic ulcers in sampled patients. Of the total sample, 1377 (19.3%) had a history of peptic ulcers prior to the index date. A Chi-squared test further found that there was a significant difference in the prevalences of prior peptic ulcers between cases and controls (21.7% vs. 16.8%, *p* < 0.001). Moreover, using a conditional logistic regression analysis (conditioned on sex, age, and index year), we found that the odds ratio (OR) of prior peptic ulcers for cases was 1.37 (95% confidence interval [CI]: 1.21~1.54, *p* < 0.001) compared to controls. Furthermore, the adjusted OR for cases was 1.40 (95% CI: 1.24~1.57) in comparison to controls after adjusting for hypertension and diabetes.

Table 3 presents a analysis of prior peptic ulcers stratified by age group. The results revealed that younger groups demonstrated higher ORs for prior peptic ulcers among cases than controls. In particular, the adjusted OR in cases < 30 years old was as high as 1.65 (95% CI = 1.25~2.19; *p* < 0.001) compared to controls. However, we failed to observe an association of acute appendicitis with peptic ulcers in the ≥ 60-year age group (adjusted OR = 1.19, 95% CI = 0.93~1.52).

Table 4 shows the further analyses of association between acute appendicitis and prior peptic ulcers by the presence of gastric ulcers, duodenal ulcers, *H. pylori*-associated ulcers or NSAIDs-associated ulcers. The findings consistently indicated that acute appendicitis was associated with *H. pylori*-associated peptic ulcers (adjusted OR = 1.59, 95% CI = 1.36~1.86), NSAID-associated peptic ulcers (adjusted OR = 2.18, 95% CI = 1.75~2.72), gastric ulcers (adjusted OR = 1.41, 95% CI = 1.22~1.64), or duodenal ulcers (adjusted OR = 1.27, 95% CI = 1.05~1.54).

Additionally, the sensitivity analysis of the association between acute appendicitis and prior peptic ulcers is presented in Table 5. It shows that acute appendicitis is consistently and significantly associated with prior peptic ulcers, even including cases who have received only one, two, or ≥3 peptic ulcer diagnoses prior to the index date (adjusted ORs were 1.82, 1.40, and 1.52 for cases who have received one, two, or ≥3 peptic ulcer diagnoses prior to the index date, respectively). The acute appendicitis is also significantly related with prior peptic ulcers, even including cases who received confirmed peptic ulcer diagnoses after receiving endoscopy (adjusted ORs was 1.79).

## Discussion

This case-control study revealed that 21.7% of hospitalized patients due to acute appendicitis had a history of peptic ulcers. A conditional logistic regression analysis showed that previous peptic ulcers were significantly associated with acute appendicitis (OR = 1.37, 95% CI = 1.21~1.54).

Mechanisms affecting the association between peptic ulcers and acute appendicitis remain unclear. The high prevalence of prior peptic ulcers in patients with acute appendicitis can possibly be explained by an autonomic imbalance and spasms of the neuromusculature at the appendicocecal junction. Previous studies indicated that the mechanism of dyskinesia of the pylorus (pylorospasms) is similar to dysfunction at the appendicocecal junction, because both of them share the same sympathetic nerve origin^8^,^10^. One recent animal study even demonstrated that antral ulcers significantly destroy the innervation between the intramural descending neuron and pyloric sphincter muscles, which result in pylorospasms and gastric emptying malfunction^9^. Some studies also reported that pylorospasms occur in 27% of duodenal ulcer and 89% of gastric ulcer patients^10^,^11^,^16^. Furthermore, studies found that appendicitis was significantly associated with duodenal ulcers, but not with *Helicobacter pylori*^10^,^11^,^17^,^18^,^19^. Our findings also suggest that peptic ulcers themselves were associated with acute appendicitis.

Bacteria are nowadays considered to play the most important role in appendiceal inflammation, in contrast to obstruction of the appendix^20^,^21^,^22^. Previous studies suggested that *Fusobacterium nucleatum/necrophorum* infection was responsible for the majority of causes of acute appendicitis^22^,^23^. Overgrowth of intragastric bacteria including *Fusobacterium* spp. is due to hypochlorhydria after long-term use of proton pump inhibitors (PPIs)^24^. Thus, peptic ulcers with long-term use of gastric acid secretory inhibitors could be potential risk factors for acute appendicitis.

However, the present study failed to observe an association between acute appendicitis and peptic ulcers in the elderly (≥60 years old) (OR = 1.18, 95% CI = 0.92~1.25). Previous studies suggested that acute appendicitis in the elderly is mainly due to lumen obstruction by residual stools or undigested food, which contribute to degenerative changes in the elderly appendix and poor colon peristalsis with age, or are secondary to frequent medications or comorbidities in the elderly^25^,^26^,^27^. According the anatomical and physiological changes in the elderly, the pathogenesis of appendiceal inflammation differs from that in young adults^25^,^26^.

This study has several strengths. First, a population-based dataset with a large sample size was used to explore the relationship between of acute appendicitis and peptic ulcers. The large sample size afforded a considerable statistical advantage in detecting real differences between cases and controls. Second, we believe that the diagnoses of peptic ulcers had very high validity because they were made by certified gastroenterologists after performing panendoscopy.

Nevertheless, results of this study need to be seen in the light of several limitations. The first limitation is that the LHID2005 provides no information on dietary fiber consumption, histology, or the existence of fecaliths in appendectomy specimens^28^. Second, physicians do not routinely identify the bacterial species in non-complicated appendicitis cases in Taiwan. This may have influenced the pathogenesis of acute appendicitis. Third, the NHI program in Taiwan was initiated in 1995, so the dataset used in the present study only allowed us to trace medical utilization from 1996 to 2013. Therefore, we could not exclude those patients who had undergone appendectomy before 1996. Therefore, it is very possible that some of the controls in this study had undergone appendectomy before 1996. However, if such a bias exists in the data, the results of our analysis would be biased towards the null. Finally, the severity of peptic ulcers could not be determined from the registry.

Despite the aforementioned limitations, this study demonstrated a potential association between acute appendicitis and peptic ulcers. We suggested that clinicians be alert in suspecting acute appendicitis for abdominal pain in patients with a history of peptic ulcers. Additionally, clinicians can provide instructions for patients with a history of peptic ulcers to seek medical services as soon as possible if they have severe abdominal pain. Nevertheless, further studies are suggested to explore the potential pathophysiology mechanisms for the relationship between acute appendicitis and peptic ulcers.

## Additional Information

**How to cite this article**: Tsai, M.-C. *et al.* Acute Appendicitis Is Associated with Peptic Ulcers: A Population-based Study. *Sci. Rep.* **5**, 18044; doi: 10.1038/srep18044 (2015).

## Figures and Tables

**Table 1:** Demographic characteristics of patients with acute appendicitis and controls (*n* = 7148).

Variable	Patients with acute appendicitis *n* = 3574		Controls *n* = 3574		
	Total no.	Column %	Total no.	Column %	*p* value
Male	1851	51.8	1851	51.8	>0.999
Age group					>0.999
18~29	924	25.9	924	25.9	
30~39	776	21.7	776	21.7	
40~49	661	18.5	661	18.5	
50~59	583	16.3	583	16.3	
60~69	296	8.3	296	8.3	
Urbanization level					0.951
1 (most)	1146	32.1	1132	31.7	
2	993	27.8	995	27.8	
3	547	15.3	571	16.0	
4	480	13.4	476	13.3	
5 (least)	408	11.4	400	11.2	
Monthly income					0.067
NT$0~15,840	1582	44.3	1490	41.7	
NT$15,841~25,000	1143	32.0	1171	32.8	
≥NT$25,001	849	23.7	913	25.5	
Geographic region					0.132
Northern	1738	48.6	1758	49.2	
Central	798	22.3	815	22.8	
Southern	932	26.1	926	25.9	
Eastern	106	3.0	75	2.1	
Co-morbidities					
Hypertension	631	17.7	712	19.9	0.014
Diabetes	286	8.0	333	9.3	0.048
Hyperlipidemia	484	13.5	489	13.7	0.863
Congestive heart failure	58	1.6	72	2.0	0.215
Chronic renal failure	70	2.0	74	2.1	0.736
Cirrhosis	116	3.3	93	2.6	0.106
Malignancy	101	2.8	101	2.8	1.000

The exchange rate in 2011 was US$1 ≈ New Taiwan dollar (NT$)31.

**Table 2:** Crude and adjusted odds ratios (ORs) and 95% confidence intervals (CIs) for acute appendicitis of sampled patients.

Presence of prior peptic ulcers	Total study sample *N* = 7148		Patients with acute appendicitis *n* = 3574		Controls *n* = 3574	
	No.	%	No.	%	No.	%
Yes	1377	19.3	775	21.7	602	16.8
Odds ratio (95% CI)	—		1.37*** (1.21~1.54)		1.00	
Adjusted OR (95% CI)	—		1.40*** (1.24~1.57)		1.00	

The adjusted OR was calculated by a conditional logistic regression which was adjusted for hypertension and diabetes.

***p < 0.001, **p < 0.01, *p < 0.05.

**Table 3:** Crude and adjusted odds ratios (ORs) and 95% confidence intervals (CIs) for acute appendicitis of sampled patients by age group.

Presence of prior peptic ulcers	Age group (years)					
	18~29		30~59		≥60	
	Patients with acute appendicitis *n* (%)	Controls *n* (%)	Patients with acute appendicitis *n* (%)	Controls *n* (%)	Patients with acute appendicitis *n* (%)	Controls *n* (%)
Yes	140 (15.2)	90 (9.7)	442 (21.9)	340 (16.8)	193 (30.6)	172 (27.3)
Odds ratio (95% CI)	1.66*** (1.25~2.19)	1.00	1.38*** (1.18~1.62)	1.00	1.18 (0.92~1.50)	1.00
Adjusted OR (95% CI)	1.65*** (1.25~2.19)	1.00	1.41*** (1.20~1.65)	1.00	1.19 (0.93~1.52)	1.00

The adjusted OR was calculated by a conditional logistic regression which was adjusted for hypertension and diabetes.

***p < 0.001, **p < 0.01, *p < 0.05.

**Table 4:** Crude and adjusted odds ratios (ORs) and 95% confidence intervals (CIs) for acute appendicitis of sampled patients by gastric ulcer, duodenal ulcer, H. pylori-associated peptic ulcer, and NSAIDs-associated peptic ulcer.

Outcomes	Patients with acute appendicitis *n* = 3574		Controls *n* = 3574	
	No.	%	No.	%
Presence of prior gastric ulcer	464	13.0	354	9.9
Odds ratio (95% CI)	1.36*** (1.17~1.57)		1.00	
Adjusted OR (95% CI)	1.41*** (1.22~1.64)		1.00	
Presence of prior duodenal ulcer	256	7.2	210	5.9
Odds ratio (95% CI)	1.24* (1.02~1.49)		1.00	
Adjusted OR (95% CI)	1.27* (1.05~1.54)		1.00	
Presence of prior H. pylori-associated peptic ulcer	449	12.6	305	8.5
Odds ratio (95% CI)	1.54*** (1.32~1.80)		1.00	
Adjusted OR (95% CI)	1.59*** (1.36~1.86)		1.00	
Presence of prior NSAIDs-associated peptic ulcer	255	7.1	128	3.6
Odds ratio (95% CI)	2.07*** (1.66~2.57)		1.00	
Adjusted OR (95% CI)	2.18*** (1.75~2.72)		1.00	

The adjusted OR was calculated by a conditional logistic regression which was adjusted for hypertension and diabetes.

***p < 0.001, **p < 0.01, *p < 0.05.

**Table 5:** Sensitivity analysis.

Presence of prior peptic ulcers	Patients who received ≥ 1 peptic ulcer diagnosis		Patients who received ≥2 peptic ulcer diagnoses		Patients who received ≥3 peptic ulcer diagnoses		Patients who received peptic ulcer diagnoses after receiving endoscopy	
	Patients with acute appendicitis *n*, %	Controls *n*, %	Patients with acute appendicitis *n*, %	Controls *n*, %	Patients with acute appendicitis *n*, %	Controls *n*, %	Patients with acute appendicitis *n*, %	Controls *n*, %
Yes	1052 (29.4)	688 (19.3)	775 (21.7)	602 (16.8)	632 (17.7)	463 (13.0)	569 (15.9)	352 (9.9)
Crude OR (95% CI)	1.75*** (1.57~1.95)	1.00	1.37*** (1.21~1.54)	1.00	1.44*** (1.27~1.64)	1.00	1.73*** (1.50~2.00)	1.00
Adjusted OR (95% CI)	1.82*** (1.63~2.04)	1.00	1.40*** (1.24~1.57)	1.00	1.52*** (1.33~1.73)	1.00	1.79*** (1.55~2.07)	1.00

The adjusted OR was calculated by a conditional logistic regression which was adjusted for hypertension and diabetes.

*p < 0.001.
